# Protein signatures of oxidative stress response in a patient specific cell line model for autism

**DOI:** 10.1186/2040-2392-5-10

**Published:** 2014-02-10

**Authors:** Andreas G Chiocchetti, Denise Haslinger, Maximilian Boesch, Thomas Karl, Stefan Wiemann, Christine M Freitag, Fritz Poustka, Burghardt Scheibe, Johann W Bauer, Helmut Hintner, Michael Breitenbach, Josef Kellermann, Friedrich Lottspeich, Sabine M Klauck, Lore Breitenbach-Koller

**Affiliations:** 1 Division of Molecular Genome Analysis, Deutsches Krebsforschungszentrum (DKFZ), Im Neuenheimer Feld 580, 69120 Heidelberg, Germany; 2 Department of Cell Biology, University of Salzburg, Hellbrunnerstr. 34, 5020 Salzburg, Austria; 3 Department of Child and Adolescent Psychiatry, Psychosomatics and Psychotherapy, J.W. Goethe University, Deutschordenstr. 50, 60528 Frankfurt am Main, Germany; 4 Department of Dermatology, General Hospital Salzburg/PMU, Müllner-Hauptstr. 48, 5020 Salzburg, Austria; 5 Max-Planck-Institute of Biochemistry, Protein Analysis Group, Am Klopferspitz 18, 82152 Martinsried, Germany

**Keywords:** Autism spectrum disorder, RPL10, Translation, Protein expression, Redox-sensitive protein signature, Oxidative stress response, Energy metabolism

## Abstract

**Background:**

Known genetic variants can account for 10% to 20% of all cases with autism spectrum disorders (ASD). Overlapping cellular pathomechanisms common to neurons of the central nervous system (CNS) and in tissues of peripheral organs, such as immune dysregulation, oxidative stress and dysfunctions in mitochondrial and protein synthesis metabolism, were suggested to support the wide spectrum of ASD on unifying disease phenotype. Here, we studied in patient-derived lymphoblastoid cell lines (LCLs) how an ASD-specific mutation in ribosomal protein RPL10 (RPL10[H213Q]) generates a distinct protein signature. We compared the RPL10[H213Q] expression pattern to expression patterns derived from unrelated ASD patients without RPL10[H213Q] mutation. In addition, a yeast *rpl10* deficiency model served in a proof-of-principle study to test for alterations in protein patterns in response to oxidative stress.

**Methods:**

Protein extracts of LCLs from patients, relatives and controls, as well as diploid yeast cells hemizygous for *rpl10*, were subjected to two-dimensional gel electrophoresis and differentially regulated spots were identified by mass spectrometry. Subsequently, Gene Ontology database (GO)-term enrichment and network analysis was performed to map the identified proteins into cellular pathways.

**Results:**

The protein signature generated by RPL10[H213Q] is a functionally related subset of the ASD-specific protein signature, sharing redox-sensitive elements in energy-, protein- and redox-metabolism. In yeast, *rpl10* deficiency generates a specific protein signature, harboring components of pathways identified in both the RPL10[H213Q] subjects’ and the ASD patients’ set. Importantly, the *rpl10* deficiency signature is a subset of the signature resulting from response of wild-type yeast to oxidative stress.

**Conclusions:**

Redox-sensitive protein signatures mapping into cellular pathways with pathophysiology in ASD have been identified in both LCLs carrying the ASD-specific mutation RPL10[H213Q] and LCLs from ASD patients without this mutation. At pathway levels, this redox-sensitive protein signature has also been identified in a yeast *rpl10* deficiency and an oxidative stress model. These observations point to a common molecular pathomechanism in ASD, characterized in our study by dysregulation of redox balance. Importantly, this can be triggered by the known ASD-RPL10[H213Q] mutation or by yet unknown mutations of the ASD cohort that act upstream of RPL10 in differential expression of redox-sensitive proteins.

## Background

More than 1% of the general population display ASDs (autism spectrum disorders) which encompass early childhood autism, Asperger-Syndrome and pervasive-developmental-disorders not-otherwise-specified [1]. ASD patients are characterized by a clinically assessed triad of symptoms of impairments in reciprocal social interaction and communication, and of restricted interests and/or repetitive behaviors [2]. The heritability of ASD is estimated to range between 37% to 80% for the disorder [3-5] depending on sample size and structure of the studies. Genetic studies indicate that a combination of common but lowly penetrant and rare but highly penetrant variants can explain a large proportion of the susceptibility for ASD [6,7]. Although single gene disorders with increased risk for ASD, ASD-specific mutations and chromosomal copy number variations are estimated to explain up to 20% of the liability for the disorder [8], the majority of cases, as well as the underlying biological pathomechanisms, remain elusive. This is in part due to the very wide difference of penetrance of each one of the three autistic core impairments recognizable in autistic patients [9]. Therefore, autism research now has a focus on the investigation of common cellular pathophysiologies, within which individual, patient specific malfunctions would generate a personalized autistic phenotype. So far, results obtained from genetic, functional and biomarker screens have been gathered to assemble the heterogeneous risk factors into pathways affected in ASD [10,11]. Among these are pathways of energy metabolism, neurotransmitter signaling and/or oxidative stress response and protein synthesis in the context of synaptic plasticity [12-15]. These studies confirmed that the functional impact of genetic variants is not only determined by the known functional role of the gene product, but also by its network context [16]. Thus, studies investigating putatively causative genetic variants in cases of otherwise undefined genetic status may prove beneficial for understanding malfunctions in cellular pathways altered in ASD [17].

We have previously characterized the genetics of two rare X-linked, recessive amino acid substitutions in the *RPL10* gene (Ribosomal Protein of the Large Ribosomal Subunit 10) co-segregating with ASD in three unrelated German families [18,19]. At present, only a few other reports on the genetics of human RPL10 are available. Gong *et al*. did not identify any additional *RPL10* mutations when screening 141 patients, which could probably be attributed to the low frequency of *RPL10* mutations [20]. At the level of mRNA expression, Gong *et al*. reported no differential regulation of RPL10 in ASD [20]. This is supported by our showing that the *RPL10* mutations neither affect X-chromosomal inactivation nor alter the overall mRNA expression levels of *RPL10*[19]. However, a suggestive etiological role of RPL10 is emerging from genome-wide studies: for example, Piton *et al*. identified a novel exonic silent mutation C > T [A211A] [21] of yet unknown pathological impact. Silent mutations have been reported to influence translation kinetics and protein folding [22,23]. While the pathophysiological role of the silent RPL10 C > T [A211A] mutation is not known, we note that the corresponding amino-acid position falls within a RPL10 C-terminal sequence tract delineated by the RPL10[L206M] and the RPL10[H213Q] mutations identified in ASD patients described by us [18,19]. Piton *et al*. did not identify non-synonymous mutations in RPL10 [21]. These results are underlined by the finding that the functionality of RPL10 is readily compromised by single amino-acid mutations affecting one of the many functions of this translational regulator [24-26] rather than by changes in copy number [27]. Again, this is in agreement with both the rare occurrence of RPL10 single amino-acid mutations in ASD specific screens and the results of two genome-wide copy number variation (CNV) studies, which reported *de-novo* copy number duplication spanning RPL10 in two independent male patients but not in controls [14,28].

Collectively, our and other studies suggest that RPL10 dysfunction in ASD pathophysiology may be triggered by either altered gene dosage or loss of function within the cellular compartment of RPL10 activity. Indeed, we and others have shown in the eukaryotic model system yeast that selected mutations in *rpl10* or altered gene-dosage of *rpl10* and other ribosomal proteins selectively shift the mRNA expression profile to generate altered expression levels of distinct protein sets [24,27]. At the functional level, this is thought to be mediated by altering the role of Rpl10 during initiation of translation [29,30] and mRNA recruitment and/or in the kinetics of accommodating the incoming tRNAs during elongation of protein synthesis [26]. Interestingly, we could show that the rare RPL10 variants, L206M and H2013Q, reduce the cellular translational capacity in a yeast model, that is, decrease of translating polysomes and increase in translationally less active monosomes and free ribosomal subunits [18]. It is known that wild-type yeast cells employ this strategy in response to a change in metabolic conditions, either in response to a change in nutrient availability [31] or in response to oxidative stress [32]. While reduction of translational capacity is associated with reduction of global protein synthesis, it is also accompanied by increased expression of distinct stress response proteins [32].

Studies in peripheral cells, such as lymphoblastoid cell lines (LCL), have been successfully employed to study dysregulation of protein expression in ASD [33]. In a systematic review Rossignol and Frye reported studies in LCLs that monitored abnormalities in ASD within distinct pathways, with the strongest evidence for immune dysregulation/inflammation and oxidative stress [34]. In this study here, making use of available LCLs we investigated the effect of the human ASD-specific RPL10[H213Q] mutation [Genebank NM_006013, c.639C > G] on cellular pathophysiology, in particular on protein expression of LCLs, and performed a comparative study employing an unrelated ASD patient set.

The effect of the RPL10[H213Q] mutation on protein expression was analyzed by a comparative two-dimensional-DIGE (difference gel electrophoresis) approach studying RPL10[H213Q] mutation carriers, RPL10 wild-type relatives and unrelated controls. Comparing RPL10[H213Q] induced proteomic changes to protein patterns present in unrelated ASD patients without any mutations in RPL10, we did not find individually overlapping proteins, but rather identified a common pattern among the differentially expressed proteins related to the response to oxidative stress. Finally, in a proof of principle study we aimed at testing the hypothesis that *rpl10* deficiency induces a protein expression pattern reminiscent of an oxidative stress response. When using a yeast model system, we obtained evidence that indeed a gene-dosage reduction of RPL10 generated a protein signature, which was overlapping with the pattern observed in wild-type cells subjected to oxidative stress.

This approach has allowed us to assess if and how the proteomic network is altered by the autism-associated RPL10[H213Q] mutation [Genebank: NM_006013, c.639C > G] and to which degree the resulting proteomic signature resembles alterations in protein expression present in the general ASD population without RPL10 mutations. Finally we are able to discuss our data in the context of substantial literature reports on peripheral markers of ASD pathophysiology [34] and references therein.

## Methods

### Selection of patients and controls

LCLs from RPL10[H213Q] mutation carrier families 277 and 440 [18,19] were available for the two male index patients (hemizygous) and the mothers (heterozygous), an unaffected carrier sister of family 440 (heterozygous) and an unaffected wild-type half-brother of family 277. Two randomly selected unrelated controls (male and female) were chosen as wild-type controls for this RPL10 mutation study. For the study of ASD-specific protein patterns LCLs from 10 male RPL10 wild-type ASD patients (ASD setup) were matched for ethnicity (Caucasian) and diagnosis (International Classification of Diseases 10^th^ revision (ICD-10): F84.0 with IQ >65) to the index patients of families 277 and 440 [see Additional file 1: Table S1]. Ten cell lines from male uncharacterized Caucasian control individuals with RPL10 wild-type were used as controls (CTRL setup). RPL10 mutational status was confirmed by direct sequencing. LCLs had been established as published in Neitzel [35].

The investigation conformed to the principles outlined in the Declaration of Helsinki. All subjects or caregivers provided written informed consent for the participation in this study and publication of this report which had been approved by the German local institutional ethics committees in Frankfurt (Fachbereich Medizin der Johann Wolfgang Goethe-Universität Frankfurt am Main, Ethik-Kommission) and Heidelberg (Ethikkommission I der Medizinischen Fakultät Heidelberg).

### Growth conditions for lymphoblastoid cell lines

Viable cells (1 × 10^6^) from a stationary phase culture were inoculated into 5 ml RPMI medium supplied with 10% fetal bovine serum (FBS), 100 U/ml penicillin, 100 μg/ml streptomycin, 2 mM L-glutamine (all from GIBCO®, Life Technologies, Paisley, UK). Volume was doubled each time cultures reached a density of 1 × 10^6^ cells/ml. Cells were harvested at a final density of 1 × 10^6^ cells/ml in 40 ml by centrifugation at 700 × g for five minutes and washed twice with PBS.

### Yeast growth conditions

Yeast strains BY4743 (wild type (WT); genotype: *MATa/MAT*α *his3*Δ*0/his3*Δ*0; leu2*Δ*/leu2*Δ*0; met15*Δ*0/MET15; LYS2/lys2*Δ*0; ura3*Δ*0/ura3*Δ*0*) and *rpl10* deficient strain (BY4743; genotype: *MATa/MAT*α *his3*Δ *0/his3*Δ*0; leu2*Δ */leu2*Δ*0; met15*Δ*0/MET15; LYS2/lys2*Δ*0; ura3*Δ*0/ura3*Δ*0; rpl10::kanMX4/RPL10*) (all EUROSCARF, Frankfurt, Germany) were grown in 100 ml synthetic complete media with a starting concentration of OD_600_ = 0.1. Oxidative stress was induced by applying 0.8 mM H_2_O_2_ after the cells had reached an OD_600_ of 0.4. Cells were harvested at an OD_600_ = 0.7 by centrifugation at 1,500 × g for five minutes and washed twice with H_2_O.

### Protein preparation

Protein preparation was performed at 4°C. LCL pellets were resuspended in 750 μl DIGE lysis buffer (7 M urea, 2 M thiourea, 30 mM Tris pH 8.5 and 4% 3-[(3-cholamidopropyl)dimethylammonio]-1-propanesulfonate (CHAPS), all from Roth, Karlsruhe, Germany) and subjected to five successive freeze and thaw cycles. Yeast cell pellets were resuspended in 1 ml DIGE lysis buffer for yeast (9 M urea, 4% CHAPS, 30 mM Tris pH 8.8, all from Roth) and mechanically lysed using 0.45 mm acid-washed glass beads. The cytosolic protein fractions from LCLs or yeast cells were purified by centrifugation at 13,000 × g for 20 minutes. The protein concentration of the supernatant was measured using the Protein Bradford Assay (Biorad, Munich, Germany).

### Two-dimensional difference gel electrophoresis

Two-dimensional-DIGE experiments were performed as previously published [36]. LCL and yeast protein extracts were processed the same way using the DIGE minimal labeling kit (GE-Healthcare, Uppsala, Sweden) according to the manufacturer’s recommendation. All individual samples were cyanine 5 (Cy5)-labelled and compared to a Cy3-labelled equimolar mixture of all samples (internal standard). Gels were scanned at a resolution of 100 μm on the Typhoon 9200 Variable Mode Imager (GE-Healthcare). DeCyder Software version 5.0 (GE-Healthcare) was used for spot detection and primary analyses; only spots with an area larger than 400 pixels and an intensity slope smaller than 2 were included. At least 20 spots per gel were matched manually and an additional 20 spots per gel were verified by manual inspection after automated spot matching. On average, 1,200 spots per gel were successfully matched and included in further calculations. The relative volume of each individual spot was normalized against the respective internal standard. T-test on the log2 ratios (sample/internal standard) comparing the two groups was performed. For further statistical analyses ‘R’ software version 2.15 was used.

### Selection of spots of interest for identification

Spots of interest were defined based on two criteria: first, candidate spots that were differentially regulated and second, spots that had a variance which was different between the two analyzed groups (this analysis was only performed in the ASD versus CTRL setup).

To identify candidates relevant to the ASD etiology including also the ones with small effects we decided to focus on T-test *P*-values only including spots regulated below the common threshold of 1.96 to 2 fold standard deviations.

After excluding the spots where the variance was similar for both (that is, the patient and control set), we focused on those spots where the variance was either larger or smaller between the two biological groups. We were also interested in minor changes and, thus, applied F-test statistics at low threshold (F-test *P*-value 0.1).

In summary, protein spots were selected for tandem mass spectroscopy (MS/MS) identification if: 1) the *P*-value of the students T-test was below 0.05; or 2) if an altered variance was detected (F-test *P*-value <0.10); and 3) if the protein spot was visible on a preparative two-dimensional gel.

### Protein identification and validation

For two-dimensional-preparative gels 500 μg of total protein were used and Coomassie stained following published protocols [37]. Picked protein spots were sent to the Max Planck Institute, Department of Biochemistry, Martinsried, Germany and identified by MS/MS.

### Computational analysis

All statistical calculations were performed using DeCyder 5.0 or ‘R’ 2.15. Hierarchical cluster analysis was performed by applying the heatmap.2() function from the R-package ‘limma’. Spots to be included in the cluster analysis were selected based on their *P*-values in the Bayes linear model predicting the biological groups using the R function lmfit(). Three models were visualized including candidates with *P*-values ≤ 0.05, ≤0.03 or ≤0.01. Best clustering was observed when selecting spots with a Bayesian model *P*-values ≤ 0.05 for ASD versus controls and ≤ 0.03 for RPL10[H213Q] mutation carriers versus non-carriers.

Network analysis was performed using Cytoscape version 2.8.2 [38] with GeneMania plug-in version 3.2 [39]. Gene Ontology (GO)-term enrichment analysis was performed using the BiNGO plug-in version 2.44 [40]. GO-terms for biological processes (GOTERM_BP_FAT) for each identified candidate (human or yeast) were retrieved from the Database for Annotation, Visualization and Integrated Discovery (DAVID) [41].

The selection of the functional sub-networks was based on key terms searched within the list of retrieved GO-terms. Candidates were included in the network named ‘Energy metabolism’ if they were associated with a GO-term including the words ‘energy’ or ‘glycolysis’. For ‘redox metabolism’ we applied the search terms ‘oxidative stress’, ‘reduction’, ‘oxidation reduction’ or ‘peroxide’. The ‘Protein and mRNA metabolism’ network was defined by the search terms ‘mRNA binding’, ‘mRNA transport’, ‘mRNA degradation’, ‘protein metabolism/catabolism/anabolism’, ‘protein folding’, ‘translation’, ‘protein localization’, ‘peptidase activity’, ‘ER’, ‘endoplasmatic reticulum’ or ‘protein catabolism’. A list of the retrieved candidates and selected GO-terms is available in Additional file 2: Table S7. We manually added *RPL10* to each list of genes if it was not a member by itself.

Selected candidates of the respective sub-networks were analyzed and visualized based on co-expression using the GeneMania plug-in mining expression data from the default 20 out of 161 databases for gene-expression available within GeneMania. For details see [42].

## Results

The impact of an ASD-associated RPL10 mutation on differential protein expression was investigated comparing LCLs of carriers of a RPL10[H213Q] mutation to wild-type RPL10 carriers including two unrelated healthy controls [see Additional file 1: Table S1 and Figure S1]. RPL10 mutant carriers were compared to RPL10 wild-type carriers disregarding ASD diagnosis to identify protein expression patterns related to the mutation and not to the ASD itself.

### Two-dimensional difference gel electrophoresis experiments

LCLs generally showed a large variance in protein expression, which can be attributed to the clonality effects during transformation as described in [43]. However, within the set of all RPL10[H213Q] mutation carriers 62 significantly regulated candidate spots were observed [see Additional file 3: Table S2] of which 7 were identified using MS/MS (Table 1). Among the up-regulated candidates we identified the lipid β-oxidation related enzyme ECH1 (FC (fold-change) = 1.76, *P*-value = 0.0003) and the translation elongation factor EEF1D (FC = 1.29, *P*-value = 0.0085). Furthermore, we observed differential expression of two central glycolytic enzymes. We report a significant down-regulation of three spots of different charge identified as TPI1 (triosephosphate isomerase, all spots with a FC < −1.30 and *P* < .01) and a trend for GAPDH (glyceraldehyde 3-phosphate dehydrogenase, FC = −1.45, *P*-value = 0.0528). For TPI1 this suggests an alteration also on the posttranslational level. Additionally, a mRNA regulatory enzyme, HNRNPK (FC = −2.79, *P*-value = 0.0017), was identified (Table 1, Additional file 3: Table S2).

**Table 1:** Proteins identified by tandem mass spectrometry following two-dimensional DIGE analysis

			**RPL10 vs Ctrl**		**ASD vs CTRL**			**MS/MS results**			
**Name**^**a**^	**Full name**	**Spot No.**	**FC**	**T-test sig.**	**FC**	**T-test sig.**	**F-test sig.**	**Cov.**	**Prot. Score**	**Comment**	**Network**
*ACTB*	Actin, beta	65	1.032	>0.1	*−1.184*	*0.0127*	*0.0949*	55%	216	Confirmed twice	NA
*ALDOC*	Aldolase C, fructose-bisphosphate	57	−1.375	0.0932	*−1.277*	*0.0018*	>0.1	44%	125	Mixture with PCBP1	Energy metabolism
*ATP5A1*	ATP synthase, H + transporting, mitochondrial F1 complex, alpha subunit 1	70	1.165	>0.1	*−1.295*	*0.0289*	>0.1	32%	153	Confirmed twice; mixture with GLUD1	Energy and redox metabolism
*ATP5B*	ATP synthase, H + transporting, mitochondrial F1 complex, beta polypeptide	61	1.087	>0.1	*1.151*	*0.0073*	>0.1	16%	48	-	Energy and redox metabolism
*ATP5H*	ATP synthase, H + transporting, mitochondrial F0 complex, subunit d	77	1.036	>0.1	−1.013	>0.1	*0.0896*	67%	134	Confirmed twice	Energy and redox metabolism
**ECH1**	Enoyl Coenzyme A hydratase 1, peroxisomal	9	**1.762**	**0.0003**	−1.690	>0.1	>0.1	27%	46	-	Energy metabolism
*ECHS1*	Enoyl Coenzyme A hydratase, short chain, 1, mitochondrial	59	−1.016	>0.1	*−1.405*	*0.004*	>0.1	12%	43	Confirmed twice	NA
**EEF1D**	Eukaryotic translation elongation factor 1 delta	75	**1.285**	**0.0085**	−1.078	>0.1	*0.0429*	27%	71	-	Protein and mRNA metabolism
ENOA	Alpha-enolase	20	−1.058	>0.1	−1.068	>0.1	>0.1	33%	81	Picked as potential housekeeper	NA
*ERP29*	Endoplasmic reticulum protein 29	58	1.258	>0.1	*2.287*	*0.0024*	>0.1		1 Prot^b^	-	Protein and mRNA metabolism
***GAPDH***	Glyceraldehyde-3-phosphate dehydrogenase	18	**−1.453**	**0.0528**	−1.005	>0.1	>0.1		1 Prot^b^	-	Energy and redox metabolism
		18c	−1.061	>0.1	*1.078*	*0.0294*	>0.1	59%	191	-	
*GLUD1*	Glutamate dehydrogenase 1	70	1.165	>0.1	*−1.295*	*0.0289*	>0.1	20%	78	Confirmed twice; mixture with ATP5A1	Redox metabolism
*HNRNP A2B1*	Heterogeneous nuclear ribonucleoprotein A2/B1	71	−1.242	0.0796	*1.173*	*0.0298*	*0.0391*	58%	196	Confirmed twice	Protein and mRNA metabolism
		18b	1.008	>0.1	*1.100*	*0.0286*	>0.1	45%	104	Confirmed twice	
***HNRNPK***	Heterogeneous nuclear ribonucleoprotein K	14	**−2.792**	**0.0017**	−1.209	0.0955	*0.0003*	13%	36	-	Protein and mRNA metabolism
*HNRPDL*	Heterogeneous nuclear ribonucleoprotein D-like	80	−1.037	>0.1	1.073	>0.1	*0.0323*	19%	78	Confirmed twice	Protein and mRNA metabolism
*HSPA5*	Heat shock 70 kDa protein 5 (glucose-regulated protein, 78 kDa)	21	−1.130	>0.1	1.004	>0.1	*0.0589*	21%	94	-	Protein and mRNA metabolism
*HSPD1*	Heat shock 60 kDa protein 1	74	1.357	>0.1	*1.130*	*0.0385*	>0.1	25%	128	Picked twice; once mixture with Vimentin, once HNRNPK	Protein and mRNA metabolism
		19	−1.060	>0.1	1.025	>0.1	>0.1	30%	93	-	
*PCBP1*	Poly(rC) binding protein 1	57	−1.375	0.0932	*−1.277*	*0.0018*	>0.1	43%	96	Mixture with ALDOC	Protein and mRNA metabolism
*PGK1*	Phosphoglycerate kinase 1	72	−1.130	>0.1	*−1.118*	*0.0299*	>0.1	60%	176	Confirmed twice	Energy metabolism
		73	−1.513	>0.1	*−1.117*	*0.0326*	>0.1	45%	150	Confirmed twice	
*PRDX2*	Peroxiredoxin 2	78	1.742	>0.1	−1.088	>0.1	*0.0095*	40%	147	Confirmed twice	Redox metabolism
*PSMA1*	Proteasome (prosome, macropain) subunit, alpha type, 1	51	−1.189	>0.1	*−1.059*	*0.0483*	0.0487	58%	132	Confirmed twice	Protein and mRNA metabolism
*PSME2*	Proteasome (prosome, macropain) activator subunit 2 (PA28 beta)	63	1.115	>0.1	*−1.226*	*0.0120*	0.0526	38%	179	Confirmed twice	Protein and mRNA metabolism
*TAGLN2*	Transgelin 2	67	−1.026	>0.1	*−1.190*	*0.0220*	>0.1		1Prot^b^	-	NA
**TPI1**	Triosephosphate isomerase 1	7	**−1.350**	**0.0074**	−1.043	>0.1	>0.1	NA		Spot mapping^c^	Energy and redox metabolism
		11	**−1.430**	**0.0001**	−1.038	>0.1	>0.1	34%	101	-	
		13	**−1.436**	**0.0043**	1.015	>0.1	>0.1	95%	192	-	

^a^Official gene symbol; ^b^1Prot: only peptides corresponding to the identified protein were identified; ^c^SWISS-2DPAGE [44] Release 19.00^*^, Mapping reference gel: Acc: P60174: LYMPHOCYTE HUMAN. Proteins in **bold** have been identified in the RPL10[H213Q] versus Controls (Ctrl) experiment, proteins in *italics* in the ASD versus CTRL setup. Cov, coverage; DIGE, difference gel electrophoresis; FC, fold change; MS/MS, tandem mass-spectrometry; NA, not applicable; Prot, protein; sig, significance (*P*-values); Spot No., number assigned by the authors during the experiment.

**Figure 1:**
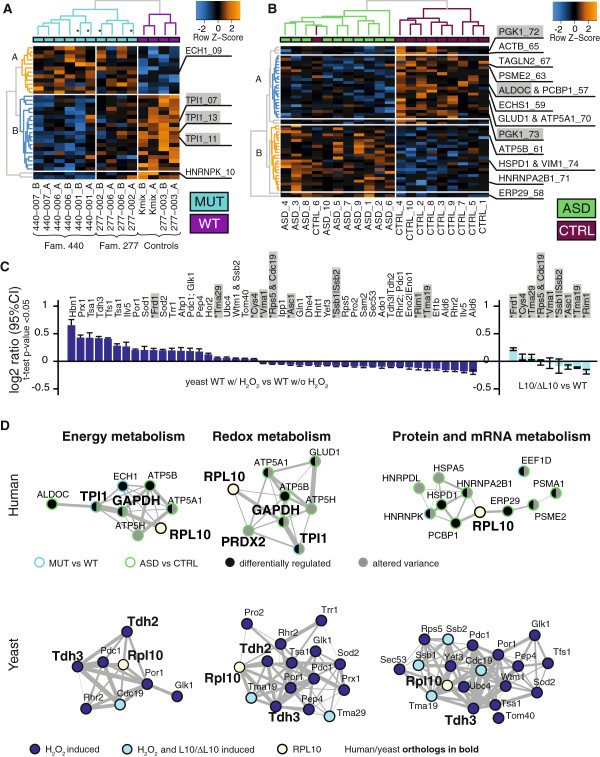
**Two-dimensional-DIGE results. (A)** Hierarchical clustering of RPL10[H213Q] mutation carriers (MUT) and wild-type allele carriers (WT) including spots with *P*-value ≤0.03 only. These 33 spots separated MUT from WT and, in addition, separately clustered families. Two distinct protein clusters **(A, B)** are visible with cluster B being more affected in family 440. Asterisks mark samples from ASD patients. **(B)** Best hierarchical clustering between ASD and controls (CTRL) was observed when selecting spots with *P*-value ≤0.05 (N = 52). Two distinct clusters are down- **(A)** and up-regulated **(B)**, respectively, in the ASD cohort. For better visualization missing values in **A** and **B** omitted during calculations are shown in black. **(C)** In yeast, proteins differentially regulated under hydrogen peroxide (H_2_O_2_) induced oxidative stress overlap with a set of proteins (marked in grey) differentially regulated in the *rpl10* deficient yeast strain L10/ΔL10 (all *P*-values ≤0.05, comparison for both conditions are to wild-type yeast). **(D)** Co-expression networks of identified candidates based on biological functions related to GO-terms. RPL10 was manually spiked into each network and is co-regulated in every module. Gene-expression data are retrieved from the GeneMania database. For details on methods and network construction see the Materials and Methods section. Symbols used in protein names in **B** and **C**: ‘&’ = a mixture of both proteins was identified; ‘|’ = MS data were not able to clearly distinguish between named protein isoforms. ASD, autism spectrum disorders; DIGE, difference gel electrophoresis; GO, Gene Ontology database; MS, mass spectroscopy.

Hierarchical cluster analysis using all detected protein spots in RPL10[H213Q] carriers did not distinguish carriers from non-carriers. However, selecting spots that were contributing to a Bayesian linear model (BLM) predicting mutation status with a significance of *P*-value ≤0.03 allowed correct clustering of individuals according to mutation status. This criterion was fulfilled by 33 spots, among them TPI1, ECH1 and HNRNPK. GAPDH was slightly above the threshold (BLM *P*-value = 0.0340) and, thus, was excluded but should still be considered as a biologically relevant target of differential protein expression in a RPL10[H213Q] background. In addition, using cluster analysis we were able to demonstrate that the protein profile of RPL10[H213Q] carriers is distinct from wild-type carriers, as the unaffected brother of family 277 clearly maps to the cluster representing the controls. Moreover, the protein signatures of the mutation carriers themselves fall into two distinct groups, representing the individual families’ genetic background (Figure 1A). We conclude that BLM cluster analysis is able to both separate carriers from non-carriers as well as to report similarities within the carriers of the two families.

To distinguish between RPL10[H213Q]-derived changes and ASD-specific variations independent of the RPL10 mutation, we advanced to proteome analysis of LCLs derived from 10 male patients, all with a comparable ASD diagnosis and similar IQ and 10 unrelated randomly selected male controls [see Additional file 1: Table S1 and Figure S1]. Of the 1,215 detected protein spots 1,195 passed the quality criteria. We investigated protein patterns with respect to change in protein expression level (T-test *P*-value ≤0.05) and variance of protein expression (F-test ≤0.1). Sixty-six candidates showed a significantly altered expression level of which 17 spots were identified using tandem MS/MS (Table 1), representing 15 different proteins (PCBP1, ALDOC, ERP29, ECHS1, ATP5B, PSME2, ACTB, TAGLN2, HNRNPA2B1, two isoforms, ATP5A1, GAPDH, PGK1, two isoforms, HSPD1, PSMA1, GLUD1; Table 1, Additional file 1: Figure S2, for full names see Additional file 4: Table S4). Of the more than 200 protein spots that had a differential expression variance we identified 10 proteins. Of these, four proteins had also been identified as being differentially expressed (ACTB, HNRNPA2B1, PSME2, PSMA1) and six additional proteins exclusively showed an altered variance in protein expression (ATP5H, EEF1D, HNRNPK, HNRPDL, HSPA5, PRDX2, see also Table 1 and Additional file 5: Table S3).

Clustering of individuals according to affected status was not possible using all spots. The best hierarchical clustering between ASD samples and controls was observed when selecting spots that contributed to the underlying BLM with a significance of *P*-value ≤0.05 (Figure 1B), suggesting a specific set of proteins to be etiologically relevant. Twelve of the 52 spots that fulfilled these criteria had already been identified in the differentially regulated set mentioned above. Again, a distinct protein signature emerged which is differentiating cases and controls. However, we also note that one of the control individuals has a protein profile reminiscent of the ASD patient set (Figure 1B).

Candidate proteins (N = 21) collectively identified in the ASD versus CTRL set mapped to etiopathologically relevant pathways previously described in neurodevelopmental disorders: energy metabolism (ALDOC, ATP5A1, ATP5B, ATP5H, ECHS1, GAPDH, PGK1, GLUD1), mRNA and protein metabolism (EEF1D, ERP29, HSPA5, HNRNPA2B1, HNRPDL, HNRNPK, HSPD1, PCBP1, PSMA1, PSME2), cytoskeleton (ACTB, TAGLN2) and redox scavenger (PRDX2). Isoforms of two proteins, HNRNPA2B1 and PGK1, were detected. For example, the glycolytic enzyme PGK1 showed two spots down-regulated (FC < −1.11, and *P*-values <0.05), suggesting again an alteration at the level of post-translational modification.

Interestingly, none of the differentially expressed proteins identified in the RPL10 family cohort was overlapping with the differentially expressed proteins in the general ASD cohort. However, an overlap within the ASD cohort showing altered variance was observed. This overlapping set included HNRNPK isoforms, EEF1D and ECH1, which is the mitochondrial homolog to the peroxisomal ECHS1.

### Validation of protein signatures

In an attempt to validate our proteome results with other techniques, we used the investigated LCLs for RT-PCR of candidate mRNAs [see Additional file 6: Table S8] and Western blot analysis [see Additional file 7: Table S9]. Of the five significantly regulated candidates identified in RPL10[H213Q] versus RPL10-WT setup (ECH1, EEF1D, GAPDH, HNRNPK and TPI1), we were able to confirm increased protein expression for ECH1 (FC = 1.32; *P*-value = 0.0250 U-test), but no changes in mRNA expression. Interestingly, we identified alterations at the protein level in the RPL10[H213Q] mutation carriers of candidate proteins identified in the ASD versus CTRL setup, namely overall expression of ACTB, HNRPDL, HSPD1 and PGK1 and specific isoforms of ALDOC and ECHS1. These candidates were not significantly changed in the two-dimensional DIGE experiment but the direction of FC was comparable between the two setups.

For the ASD versus CTRL setup we were able to confirm reduction of ACTB (FC = −1.19, *P*-value = 0.041 U-test) and observed a suggestive up-regulation for ERP29 (FC = 1.26, *P*-value = 0.096) at the protein level. At the mRNA level we observed changes in the *EEF1D* and the *HNRNPK* gene-expression. At the level of protein expression in the two-dimensional DIGE setup both candidates showed an altered variance but were not differentially regulated. Our results suggest a predominant regulation of the differentially expressed proteins at the translational level. This is supported by a study of Schwanhäusser *et al*. who quantified global mammalian gene and protein expression and found that cellular abundance of proteins is predominantly controlled at the level of translation [45].

### Validation of protein signatures at functional levels

In search for validation of the candidate proteins on the functional level we started to look for shared molecular properties. The identification of a redox scavenger differing in variance (PRDX2; F-test *P*-value = 0.0095) and several differentially expressed mitochondrial enzymes (for example, ATP5A1, ATP5B, ECHS1; all T-test *P*-values <0.03) led us to the hypothesis of a redox-sensitive protein network. Indeed, a literature search revealed that 22 out of the total 25 candidates identified in this study (combining RPL10[H213Q]-regulated and ASD-specific proteins) are described as oxidative stress response proteins, or as regulators of the levels of reactive oxygen species (ROS) (for references see Additional file 1: Table S5).

To investigate a possible link between alterations in redox-sensitive proteins, energy metabolism, altered RPL10 function and change in protein expression pattern, we performed a proof-of-principle study in yeast [see Additional file 1: Figure S1]. First, we confirmed previous findings that in response to oxidative stress, exemplified by H_2_O_2_ stress, yeast wild-type cells differentially express redox scavengers, redox-sensitive enzymes as well as glycolytic enzymes [46,47] (Figure 1C and Additional file 8: Table S6). We observed regulation of yeast Tdh3 (one of the three yeast GAPDH homologues), where two isoforms were differentially expressed (FC = 1.34, *P*-value = 0.0027 and FC = −1.07, *P*-value = 0.0118). Interestingly, proteome analysis of the yeast *rpl10* deficient strain under standard conditions and not subjected to oxidative stress showed that 13 of 45 significantly (*P*-value ≤0.05) regulated protein spots were up- or down-regulated in an identical fashion as a subset of the overall set of H_2_O_2_ stress-regulated candidates in wild-type yeast cells [see Additional file 8: Table S6, Figure 1C]. We were able to identify in 9 of these 13 spots a total of 11 proteins: Asc1, Cys4, Frd1, Rim1, a mixture of Rps5 and Cdc19, Ssb1 or Ssb2, Tma19, Vma1, and Tma29 [see Additional file 4: Table S4 and Additional file 8: Table S6, Figure 1C]. In addition to the glycolytic enzyme Cdc19 (pyruvate kinase; FC = −1.04, *P*-value = 0.0189), we also noted differential regulation of a Tdh3 isoform, however with a suggestive significance only (FC = −1.03, *P*-value = 0.0777, Additional file 8: Table S6).

The overlap in glycolytic proteins in the RPL10[H213Q] mutant carriers, the ASD patient set, the oxidatively stressed yeast cells and the yeast cells with *rpl10* deficiency suggested a gene ontology association study. Indeed, a first investigation on biological processes revealed enrichment for energy metabolism, protein and mRNA metabolism, and redox metabolism [see Additional file 2: Table S7 and Additional file 1: Figure S2]. Subsequently, based on their GO-terms, we selected those candidates of related function – protein and mRNA metabolism, energy metabolism and redox metabolism – and performed a gene co-expression network analysis based on available transcriptome-data from GeneMania [48] (for details see Methods and Additional file 2: Table S7). For both yeast and human mRNA expression data, we observed that the individual networks of the selected candidates built ensembles that are co-regulated at mRNA levels with RPL10 (Figure 1D). In addition, the tight co-regulation at mRNA levels of the RPL10[H213Q] and the ASD candidate proteins strengthens the reported functional overlap between the molecular phenotypes generated by the RPL10 mutation and the ASD cohort, respectively.

## Discussion

An estimated 1 out of 110 individuals in the US is currently affected by ASD [1]. For decades up until now ASD has been clinically defined by behavioral observations following standard manuals. In spite of tremendous effort, the etiology of ASD is still unclear and large consortia have obtained genetic evidence that the majority of ASD cases do not result from simple gene or chromosomal disorder. A meta-analysis of research trends in ASD conducted by Rossignol and Frye indicates that in addition to dysfunctions of the CNS thought to be causative for the cognitive and behavioral features of ASD, pathophysiologies that transcend specific CNS function accompany most ASD cases [49]. These authors combined reports obtained in neurocognitive areas with focus on CNS malfunction (neuroimaging, neuropathology, theory of mind and genetics) with reports on the study of pathophysiological processes that could more directly result in cellular dysfunctions and the subsequent development of psychiatric disorders, such as ASD. In particular, there are well documented differences in ASD patients compared to controls in immune response, especially that of the gastrointestinal tract [50], in oxidative stress response/redox regulation and general detoxification [51], as well as in energy metabolism and associated mitochondrial function [52]. One common theme of all pathophysiological parameters reported in this meta-study is their relatively small, albeit significant, deviation from the respective level in typically developed controls. While different systemic abnormalities may accompany most ASD cases, they must be small to allow for organismal viability. In sum, this study suggests that ASD, rather than being a purely CNS disorder, displays a wide range of systemic abnormalities making the study of peripheral cells and systemic fluids an attractive target to investigate the etiology of ASD.

The results of our study mirror in several ways the documentations of Rossignol and Frye [34]. First, we report that an ASD-associated RPL10[H213Q] mutation alters protein expression patterns similar but not identical to the one observed in an unrelated ASD cohort without this mutation. This suggests a common downstream pathophysiology resulting from different unidentified upstream genetic variants. Second, the RPL10[H213Q] expression signature differentiating carriers from non-carriers, is modified by the genetic background of the carriers within families, as observed in cluster analysis. We hypothesize that distinct different protein expression levels in individuals may define distinct pathophysiological phenotypes. Furthermore, while RPL10[H213Q] heterozygous carriers, all women, and RPL10[H213Q] hemizygous carriers, all ASD cases, share common motifs in altered protein expression, the contribution of the remaining wild-type RPL10 [19] to the establishment of a healthy or subclinical phenotype of the female carriers has not been studied here. Thus, it remains to be tested if the protein signatures identified here ultimately correlate with severity of ASD as reported for mitochondrial biomarkers (for an extensive meta-review compare Rossignol and Frye [49]).

Third, the protein signatures observed in the RPL10[H213Q] mutant cohort and in the unrelated ASD cohort are characterized by both having different expression levels and containing different redox-sensitive members of identical pathways. This is in agreement with observations made by Rossignol and Frye [34], namely that depending on the study cohort, overlap is observed in same pathophysiologies.

To assess the phenomenon of oxidative stress and redox control in the context of ASD pathophysiology it is important to recall that endogenous oxidative stress results from an excess of ROS which no longer can be reduced to non-pathological levels by anti-oxidant scavenger and repair mechanisms. Ultimately, all excess ROS are generated by malfunctions in energy metabolism, primarily fueled by glycolysis in the cytoplasm and the tricarboxylic acid (TCA) cycle and oxidative phosphorylation (OXPHOS) in mitochondria. The successive breakdown of carbon fuels during glycolysis and the TCA cycle supplies electrons to the electron transport chain for mitochondrial OXPHOS to produce ATP and H_2_O. During this process leakage of electrons results in the formation of ROS, the superoxide radical (O2^–^), hydrogen peroxide (H_2_O_2_) and the hydroxyl radical (OH). If increasing amounts of ROS escape detoxification, excess oxidative power attacks lipids, proteins and DNA (reviewed in [53]). Lipid peroxidation is detrimental to the function of receptors and ion channels in all cellular membranes and, thus, also to the balance of membrane potential in the neuron [54]. Loss of control over membrane potential, in turn, has been reported to affect receptors’ function [55] and may thus lead to altered signal transduction between pre- and post-synaptic membranes. Protein oxidation leads to loss of enzyme function and/or loss of structural integrity [56]. Oxidation of DNA is a major source of somatic mutations [57]. Each of these pathologies is harmful to all cells and has been shown to have a severe negative impact on neural cells, in particular in the context of inflammation and synaptic function [54,58]. On the level of protein expression, oxidative stress is known to quantitatively and qualitatively alter mRNA translation in model systems [32,59].

Altered translation has been until now one of the best documented cellular processes executing correct synaptic plasticity by modulating density and composition of synaptic proteins, the loss of which is one of the hallmark pathologies in ASD brain tissue [60]. Our observation that altered availability of the translational regulator RPL10 generates a cellular redox-sensitive stress response signature in the yeast model, suggests that a mutation in human RPL10 could trigger a similar phenomenon in neural cells. This is supported by our observation of a redox-sensitive protein signature in LCLs of RPL10[H213Q] patients.

How then might an ASD-mutation modulate oxidative stress in the periphery and the CNS of ASD patients? First, a mutation in a translational regulator, such as the ribosomal protein RPL10, might result in unscheduled or increased recruitment of redox-sensitive proteins that are a mimic of the translational response driven by other, more upstream ASD genetic variations (for example, provided by different genetic backgrounds). Basically, in all these cases this scenario would supply the cell with a similar compromised redox microenvironment as observed in our study. If there is a related translational response for other or even most ASD cases remains to be established. Second, several affected genes in our study encode enzymes that were reported to produce or eliminate ROS and thus, when mutated lead to loss of control of ROS production associated with impaired gene expression [51]. Third, any enzyme directly active in energy metabolism, when mutated, would fulfill the criteria for an autism candidate gene, as it ultimately would favor imbalance in mitochondrial ROS production**.** This is underscored by a series of enzymes of glycolysis, the TCA cycle and OXPHOS found to be affected in neuropsychiatric diseases [61,62]. GAPDH and TPI, identified in this study to be differentially regulated in ASD, have been reported to be differentially expressed in brains of schizophrenia patients [63]. The close neighborhood of the RPL10 protein to both proteins in the energy metabolism co-expression network may thus underscore the etiological relevance of RPL10 in neurodevelopmental disorders. Finally, a protein identified as dysfunctional in ASD may be directly sensitive to cellular redox status, either by oxidative stress induced alterations in posttranslational modifications [64,65] or by shifting the equilibrium of redox-sensitive disulphide bonds in regulatory proteins [66]. The former proposal is supported by the observation of differential expression of posttranslationally altered proteins in our study, and the latter one by redox-sensitive disulphide bonds reported for ASD candidate proteins [66,67].

For a discussion of putative functional roles of individual proteins identified with the redox-sensitive proteins signature in the etiology of ASD we will focus on the ASD candidate protein RPL10, which is co-expressed with candidates in all three GO-term enriched networks identified in both human and yeast studies (Figure 1D). A second such protein is GAPDH/TDH3 which was highly connected within the co-expression networks of energy metabolism (human and yeast) and redox response (human and yeast) as well as mRNA and protein expression (yeast only, as human GAPDH is so far not GO-term annotated with translation or mRNA regulation).

The glycolytic enzyme GAPDH is a redox-sensitive key enzyme of glycolysis and together with its immediate glycolytic neighbor TPI1 (Figure 1D) has previously been reported to be differentially regulated in schizophrenia [63]. This functional interaction of human RPL10 mutations with glycolytic enzymes is confirming previous findings from yeast studies [24,25]. GAPDH and other glycolytic enzymes are receiving increasing interest as regulatory proteins in differentiation and disease [68]. It has been shown that isoforms of GAPDH and downstream glycolytic enzymes are expressed in different metabolic stages in yeast [69] as well as in cellular differentiation in mammalian cells [70]. These studies propose that posttranslational modifications of GAPDH and other glycolytic enzymes, some targeted by oxidative modification, make these glycolytic enzymes to platforms within the successive stages of energy metabolism, which can be used to switch between high and low levels of energy metabolism [69]. For example, several studies have shown, that reduction of GAPDH function reroutes the glycolytic flow to the pentose phosphate pathway, used to generate NADPH, which serves as the reducing power to restore glutathione (GSH) levels in the GSH/GSSH redox couple, the major intracellular antioxidant/detoxification species [71]. Alterations in GAPDH function, induced by mutation, altered expression level or change in posttranslational modification, may thus affect energy metabolism and redox status, which is of particular importance in high energy consuming cells like neurons.

Ribosomal protein RPL10 is also of specific interest to further studies on the etiology of ASD. So far, translational regulators described in more detail as mediating dysfunctional translation in ASD are FMRP [72] and eukaryotic initiation factor 4E, eIF4E [73]. FMRP is a translational repressor binding to and, thereby, preventing protein synthesis of synaptic mRNAs. Mutated FMRP leads to unscheduled protein synthesis at the synapse, resulting in compromised synaptic plasticity [74]. Also, mutationally altered eIF4E leads to hyperconnectivity of neural circuits due to increased synaptic protein synthesis. EIF4E is a key component of the eukaryotic translation initiation machinery, which when over expressed leads to increased expression of neuroligins, which are postsynaptic proteins that are causally linked to ASD [73]. RPL10 is a component of the translating ribosome and different modifications of RPL10 may alter different ribosome functions, for example mutations in RPL10 have been described that alter translation initiation [30] or protein expression levels [24]. At present, we do not know if there are modifications of RPL10 resulting from upstream regulators of translation, for example upstream regulators of synaptic protein expression and/or oxidative stress response. If so, it would be interesting to learn whether they are known ASD candidate proteins.

**Figure 2:**
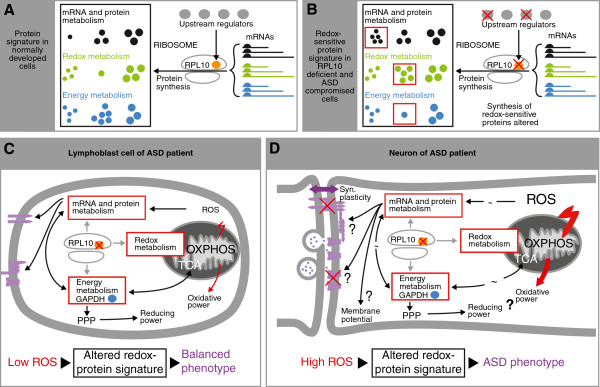
**ASD mutation-induced redox-sensitive protein signatures compromise core synaptic functions in autism. (A)** The ribosome recruits mRNAs, encoding proteins for specific pathways (black, green, blue mRNAs). Subsets of mRNAs are under the control of the translational regulator RPL10 (orange), which itself is controlled by upstream regulators (grey). This forms a protein signature characteristic for example for mRNA and protein metabolism (black), redox-metabolism (green) and energy metabolism (blue). **(B)** In the presence of (mis)functional RPL10 variants (for example, RPL10[H213Q]) or other ASD variants (red crosses) regulating RPL10 the protein signatures are selectively shifted (red rectangles) resulting in alterations (change in number of the framed protein spots) of pathway-specific redox-sensitive proteins as observed here. The altered protein signature thus indicates a response to oxidative stress elicited by the ASD mutation(s). **(C)** In peripheral tissues, for example, a lymphoblast, deficiencies in RPL10 or upstream regulators thereof alter the redox-sensitive signature in a way which in the presence of moderate ROS (reactive oxygen species) production (small red bolt) is still able to balance cellular stress response. In particular, altered expression of the glycolytic enzyme GAPDH (blue) may be employed for redox buffering by rerouting glycolysis into the PPP (pentose phosphate pathway). This will increase reductive power to balance oxidative power caused by ROS. **(D)** Under conditions of high oxygen metabolism (large red bolt) as present in neural cells, redox buffering will still support basal synaptic functions, but may no longer be able to prevent oxidative damage of lipids and proteins required for cellular fine-tuning (undulations) of synaptic functions and plasticity (purple, double headed arrow). We hypothesize that the effect of different ASD-mutation induced protein signatures would drive individual autistic phenotypes with differential failure to secure correct executing of synaptic plasticity. Question marks indicate unknown individual steps. ASD, autism spectrum disorders.

In sum, our results on differential redox-sensitive protein expression patterns in patients with RPL10[H213Q] mutations and unrelated RPL10 wild-type ASD patients, in particular in the context of alterations in energy metabolism and protein translation, have led us to formulate the following hypothesis (Figure 2). The altered redox-sensitive protein signature, as observed in peripheral cells derived from our patient sets, mirrors a cellular response to oxidative stress. We suppose that this signature is largely compatible with normal cellular function in peripheral tissues under conditions of moderate ROS levels. However, the brain is the organ with the highest oxygen consumption and metabolism [53] and, thus, we propose that in the presence of high oxygen turnover in neural cells, the altered redox-sensitive protein signatures may fail to buffer oxidative stress in energy metabolism, redox metabolism and protein metabolism. To arrive at a molecular understanding of the role of an individual, RPL10-regulated and differentially expressed redox-sensitive protein, GAPDH may serve as an example (Figure 2). GAPDH is a key glycolytic enzyme and major player in rerouting reductive power to balance increased oxidative stress. In the process of adapting to different levels of cellular oxidative stress, GAPDH may tolerate only a distinct range of change in expression level. While the altered expression level of GAPDH observed in lymphoblastoid cells may be compatible with effective redox control in this cell type, it may fail to provide the required response to the high oxygen metabolism present in a neuron. If this insufficiency in the presence of high oxygen stress either results from a loss of regulatory flexibility and/or inadequate network function as a consequence of the differentially expressed state, remains to be established.

Consequently, we propose that ASD-mutation induced altered redox-sensitive protein signatures fail to balance the high oxidative stress in the neuron. The resulting excessive oxidative damage may compromise correct functioning of synaptic proteins and membrane potential to execute synaptic plasticity.

## Conclusions

Here, we show for the first time in a patient-specific cell line model for ASD that ASD patients, either with defined or with undefined mutations at the level of protein expression, display modulated expression of redox-sensitive components in those cellular pathways that execute synaptic plasticity in the brain: energy metabolism, redox metabolism and protein metabolism. Individual differences in these expression patterns may contribute to the phenotypic continuum observed in the wide spectrum of ASD patients.

## Availability of supporting data

The data sets supporting the results of this article are included within the article and its additional files [see Additional files 1, 2, 3, 4, 5, 6, 7 and 8].

## Abbreviations

ASD: autism spectrum disorder; BLM: Bayesian linear model; CHAPS: 3-[(3-cholamidopropyl)dimethylammonio]-1-propanesulfonate; CNS: central nervous system; CTRL: control; Cy3: cyanine 3; Cy5: cyanine 5; DAVID: Database for Annotation Visualization and Integrated Discovery; DIGE: difference gel electrophoresis; FBS: fetal bovine serum; FC: fold change; GO: Gene Ontology Database; ICD-10: International Classification of Diseases Version 10; IQ: intelligence quotient; LCL: lymphoblastoid cell line; MS: mass spectrometry; MUT: mutation; OXPHOS: oxidative phosphorylation; PBS: phosphate-buffered saline; RPMI: Roswell Park Memorial Institute cell culture medium; RT-PCR: reverse transcriptase polymerase chain reaction; ROS: reactive oxygen species; TCA: tricarboxylic acid; WT: wild-type.

## Competing interests

The authors declare that they have no competing interests.

## Authors' contributions

AGC co-designed and co-conceived most experiments and together with DH performed most experiments. AGC performed statistical analyses and wrote the manuscript. AGC and DH are joint first authors. MBo performed preliminary experiments for yeast analyses. TK performed the literature search. CMF and FP recruited and diagnosed the patient sample. BS provided expertise on software for analyses of two-dimensional-DIGE data. JWB and HH provided expertise for use and the antibodies for validation experiments. JK and FL performed mass spectrometric experiments and analyzed mass spectrometry data. SW and MBr contributed to study design and provided experimental expertise. LBK and SMK conceived and designed experiments, oversaw the study, interpreted results and wrote the manuscript. SMK and LBK are joint senior authors and project co-leaders. All authors read and approved the final manuscript.

## Supplementary Material

Additional file 1 **Additional Methods.** mRNA expression and Western blot analysis. **Figure S1.** Experimental setup. **Figure S2.** Hierarchical structure of gene enrichment analysis results. **Table S1.** List of samples used in this study. **Table S5.** Literature search for redox-sensitive candidates. Click here for file

Additional file 2: Table S7 Gene enrichment analysis of identified candidate proteins. Click here for file

Additional file 3: Table S2 Two-dimensional-DIGE results of RPL10[H213Q] carriers versus wild-type allele carriers. Click here for file

Additional file 4: Table S4 List of candidates discussed in this publication. Click here for file

Additional file 5: Table S3 Two-dimensional-DIGE results of ASD versus controls. Click here for file

Additional file 6: Table S8 mRNA analysis of identified candidate proteins. Click here for file

Additional file 7: Table S9 Western blot analysis of identified candidate proteins. Click here for file

Additional file 8: Table S6 Two-dimensional-DIGE results of yeast proteomic analysis. Click here for file

## Dedication

We dedicate this paper to our late friend and mentor Annemarie Poustka.
