# Supplementary material for: Protein signatures of oxidative stress response in a patient specific cell line model for autism
Source: Mol Autism. 2014 Feb 10;5:10. doi: 10.1186/2040-2392-5-10 (PMC3931328; doi:10.1186/2040-2392-5-10)
Supplement: Additional file 1 — Additional Methods. mRNA expression and Western blot analysis. Figure S1. Experimental setup. Figure S2. Hierarchical structure of gene enrichment analysis results. Table S1. List of samples used in this study. Table S5. Literature search for redox-sensitive candidates. [file 2040-2392-5-10-S1.doc]

**Additional Information to**

**Protein signatures of oxidative stress response in a patient specific cell line model for autism**

Andreas Chiocchetti^*^, Denise Haslinger^*^, Maximilian Boesch, Thomas Karl, Stefan Wiemann, Christine M Freitag, Fritz Poustka, Burghardt Scheibe, Johann W Bauer, Helmut Hintner, Michael Breitenbach, Josef Kellermann, Friedrich Lottspeich,

Sabine M Klauck^§^ and Lore Breitenbach-Koller^§^

^*^These authors contributed equally to this work.

^§^Joint corresponding authors

**Content**

**Additional Methods 1**

**Additional Figures** **2**

**Figure S1: Experimental setup** **2**

**Figure S2: Hierarchical structure of gene enrichment analysis results** **3**

**Additional Tables** **4**

**Table S1: List of samples used in this study** **4**

**Table S2: 2D-DIGE results of RPL10[H213Q] carriers versus wild-type allele carriers**

*provided as separate excel file (Additional file 3)*

**Table S3: 2D-DIGE results of ASD versus controls**

*provided as separate excel file (Additional file 5)*

**Table S4: List of candidates discussed in this publication**

*provided as separate excel file (Additional file 4)*

**Table S5: Literature search for redox-sensitive candidates** **5**

**Table S6: 2D-DIGE results of yeast proteomic analysis**

*provided as separate excel file (Additional file 8)*

**Table S7: Gene enrichment analysis of identified candidate proteins**

*provided as separate excel file (Additional file 2)*

**Table S8. mRNA analysis of identified candidate proteins.**

*provided as separate excel file (Additional file 6)*

**Table S9. Western blot analysis of identified candidate proteins.**

*provided as separate excel file (Additional file 7)*

**Additional References** **6**

### Additional Methods

### mRNA expression

### mRNA purification was performed at 4°C using the mRNAeasy Kit (Qiagen, Hilden, Germany) according to the manufactures recommendation. Growth conditions were the same as described for protein preparation in the methods section of the main article. cDNA was generated with the RevertAid™ H Minus Reverse Transcriptase cDNA Kit (Fermentas, St. Leon-Rot, Germany) from total RNA isolated from lymphoblastoid cell lines (LCLs). Primer and Probe combinations (Additional file 8) for the respective genes were designed according to the Universal Probe Library (Roche, Mannheim, Germany) assay design center (<https://www.roche-applied-science.com/sis/rtpcr/upl>, Additional file 7). 10 ng of cDNA were used per approach. The final concentrations were: 1x Applied Biosystems Master-mix (Applied Biosystems, Darmstadt, Germany), 200nM of each primer and 100nM of the respective UPL probe. Real-time PCR was performed in a total volume of 11µl. GUSB (Glucoronidase-β) was used as reference gene. Analysis of data was performed as described in Livak and Schmittgen (2001) [1].

### Western Blot analysis

### Protein preparation was performed as described in the Methods section of the main article. 15 µg of total protein were loaded onto a 12.5% Tris-HCl-SDS-PAGE. Proteins were transferred onto a PVDF membrane in a semi-dry approach at 25mV for 60min (Biorad System, München, Germany). The membrane was blocked for 1h at room temperature (RT) in 3%BSA, 1X TBS (Tris-buffered saline, 50 mM Tris, 150 mM Sodium, pH 7.6), 0.05% Tween-20 (TBS-T). The primary antibody was diluted 1:250-5000 in blocking buffer and incubated overnight at 4°C. Performing 5 washing steps with TBS-T for 5min. each removes unbound antibodies. The second Near-Infrared-conjugated antibodies were diluted 1:10,000 in blocking buffer and incubated for 1h at RT. Five washing steps followed. Image acquisition was performed on an Odyssey system (LI-COR). For the specific primary and secondary antibodies see Additional file 8. Overall protein expression was calculated based on intensity values using ImageJ Software version 1.44p. Normalization occurred over the mean protein expression of all analyzed samples. Wilcoxon-test was performed using SPSS version 17.0.

### Additional Figures

### Figure S1 - Experimental setup


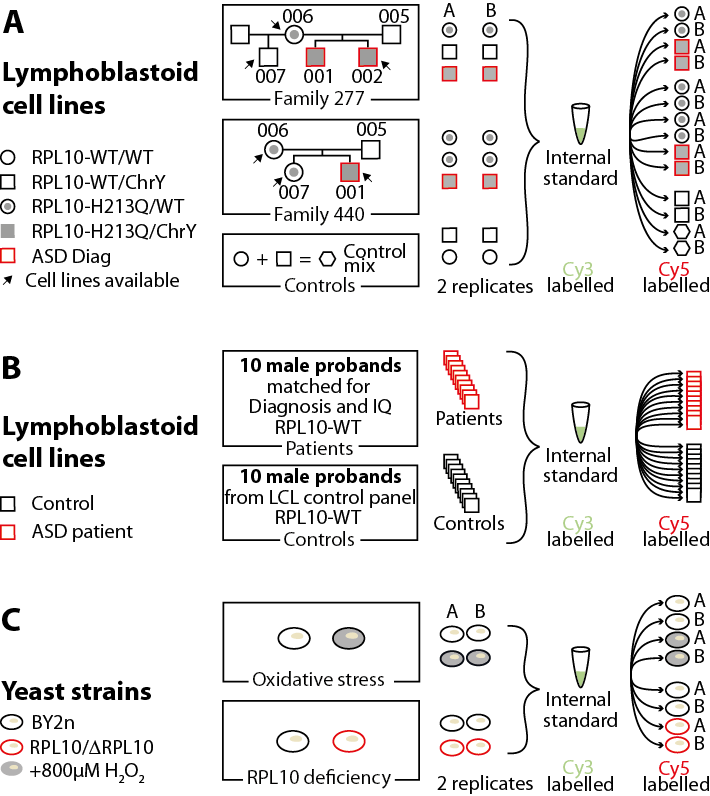


(**A**) To describe RPL10[H213Q] mutation derived differential protein expression whole protein extracts of lymphoblastoid cell lines (LCL) established from RPL10[H213Q] mutation carriers in two families are compared to a mixture of a male and a female probe (Control mix) and one related non-carrier. Arrows mark individuals with LCLs. (**B**) To assess the ASD specific expression pattern whole protein extracts from 10 male patients matched for ASD diagnosis and 10 male controls, each carrying the wild-type RPL10 sequence, are compared. (**C**) A proof of principle showing that RPL10 is related to oxidative stress was performed in a yeast model investigating a diploid wild-type (BY2n) with and without oxidative stress, and comparing BY2n to a diploid RPL10 deficient (RPL10/ΔRPL10) yeast strain under normal conditions.

In all three setups a mix of all analyzed samples was used as internal standard to reduce gel to gel variation. On each gel a single sample (labeled Cy5) was run against the respective internal standard (labeled Cy3). Biological replicates (A and B) where performed for setup **A** and **C**. For a detailed description see Methods.

### Figure S2 - Hierarchical structure of gene enrichment analysis results


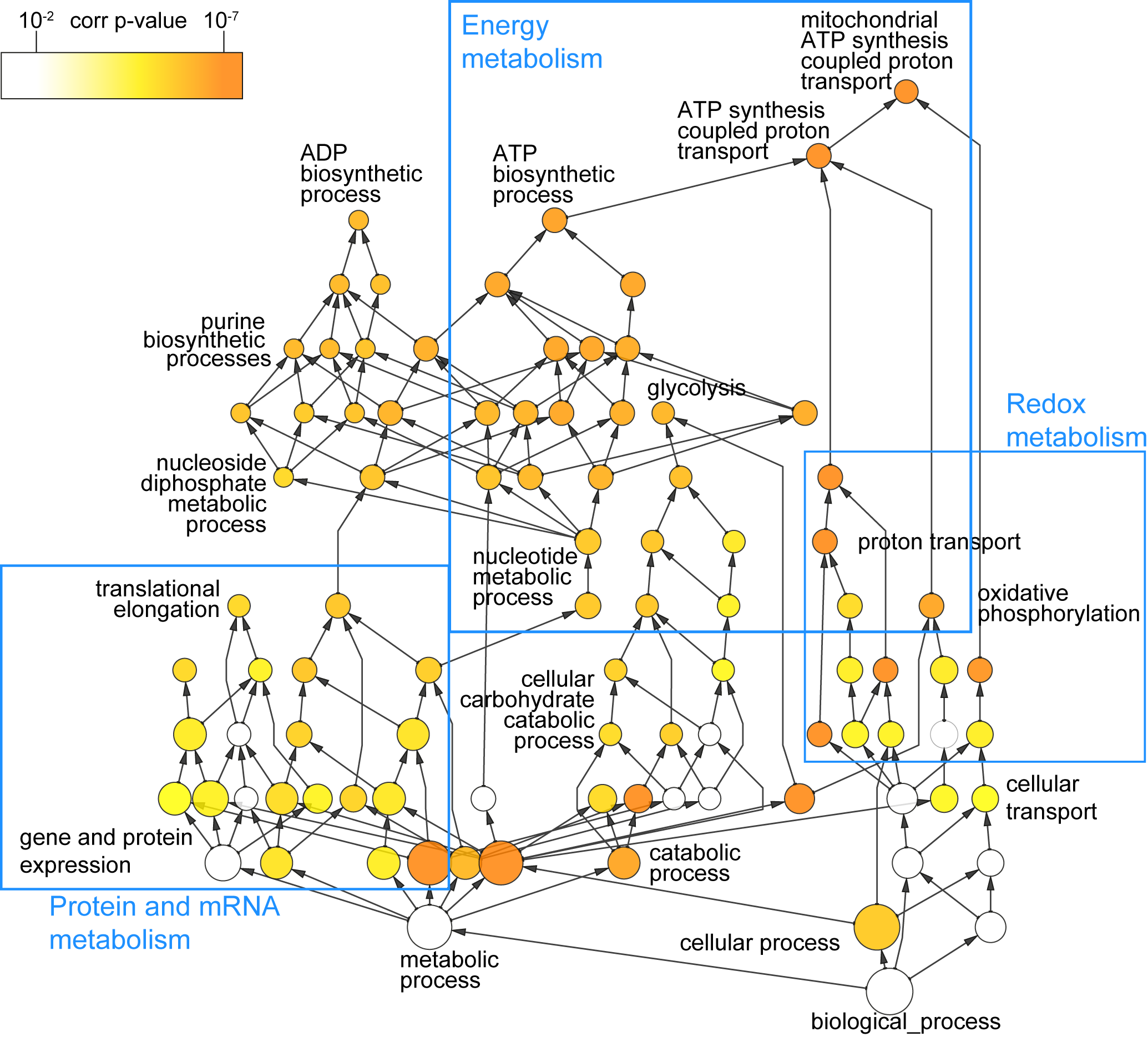


Hierarchical network of biological functions associated with the proteins identified in this study as susceptibility factors in ASD is displayed. Data and network were generated using the Cytoscape BinGO plugin with proteins listed in Additional Table S4. Every node corresponds to a specific biological process. The p-value after Bonferroni-correction is shown as color code. Blue rectangles mark the functional subgroups defined by the authors suggested by GO-term enrichment analysis. Only major biological processes are named for simplicity.

### Additional Tables

#### Table S1 - List of samples used in this study

| **ID** | **Family** | **Diag.** | **Gender** | **IQ** | **Ethnicity** | **ADI-R** | **ADOS** | **LD** |
| --- | --- | --- | --- | --- | --- | --- | --- | --- |
| 277_002 | 277 | F84.1 | Male | 69 | Caucasian | Yes | Yes | Yes |
| 277_006 | 277 | Unaff. | Female | NA | Caucasian | NA | NA | NA |
| 277_003 | 277 | Unaff. | Male | NA | Caucasian | NA | NA | NA |
| 440_001 | 440 | F84.0 | Male | 99 | Caucasian | Yes | Yes | No |
| 440_006 | 440 | Unaff. | Female | NA | Caucasian | NA | NA | NA |
| 440_007 | 440 | Unaff. | Female | NA | Caucasian | NA | NA | NA |
| CTRL_m^a^ | NA | NA | Male | NA | Caucasian | NA | NA | NA |
| CTRL_f | NA | NA | Female | NA | Caucasia | NA | NA | NA |
| AUT_1^b^ | 229 | F84.0 | Male | 76 | Caucasian | Yes | Yes | No |
| AUT_2 | 246 | F84.0 | Male | 69 | Caucasian | Yes | Yes | No |
| AUT_3 | 334 | F84.0 | Male | 100 | Caucasian | Yes | Yes | No |
| AUT_4 | 353 | F84.0 | Male | 90 | Caucasian | Yes | Yes | No |
| AUT_5 | 459 | F84.0 | Male | 88 | Caucasian | Yes | Yes | No |
| AUT_6 | 562 | F84.0 | Male | 97 | Caucasian | Yes | Yes | No |
| AUT_7 | 593 | F84.0 | Male | 94 | Caucasian | Yes | Yes | No |
| AUT_8 | 594 | F84.0 | Male | 93 | Caucasian | Yes | Yes | No |
| AUT_9 | 636 | F84.0 | Male | 87 | Caucasian | Yes | Yes | No |
| AUT_10^c^ | 660 | F84.0 | Male | 87 | Caucasian | Yes | Yes | No |
| CTRL_1 | NA | NA | Male | NA | Caucasian | NA | NA | NA |
| CTRL_2 | NA | NA | Male | NA | Caucasian | NA | NA | NA |
| CTRL_3 | NA | NA | Male | NA | Caucasian | NA | NA | NA |
| CTRL_4 | NA | NA | Male | NA | Caucasian | NA | NA | NA |
| CTRL_5 | NA | NA | Male | NA | Caucasian | NA | NA | NA |
| CTRL_6 | NA | NA | Male | NA | Caucasian | NA | NA | NA |
| CTRL_7 | NA | NA | Male | NA | Caucasian | NA | NA | NA |
| CTRL_8 | NA | NA | Male | NA | Caucasian | NA | NA | NA |
| CTRL_9 | NA | NA | Male | NA | Caucasian | NA | NA | NA |
| CTRL_10 | NA | NA | Male | NA | Caucasian | NA | NA | NA |

AUT = Proband with ASD diagnosis, CTRL= Control proband without known ASD diagnosis. M= Mother, S= Sibling, Diag. = Diagnosis applying ICD-10 criteria according to medical records, Unaff.= unaffected, ADI-R = Autism diagnostic interview- revised, ADOS = Autism Diagnostic Observation Schedule, Yes = confirms ASD diagnosis, LD = Language delay according to items in the ADI-R; NA = Not applicable.

^a^ This control is identical to CTRL_1

^b^ This patient carries a 2q37.3 deletion as previously published [2].

^c^ This patient carries a CNV deletion in the *NRXN1* gene (unpublished results).

**Table S5** - Literature search for redox-sensitive candidates

| **Gene** | **Evidence** | **Reference** | **Ref. No.** |
| --- | --- | --- | --- |
| ACTB | increased carbonylation | Poon et al., 2006 | [3] |
| ALDOC | altered PTM upon oxidative damage | Sultana et al., 2010 | [4] |
| ATP5A1 | differential PTM after exposure to NOS | Sultana et al., 2006 | [5] |
| ATP5B | Directly related to OXPHOS reaction | Leyva et al., 2003 | [6] |
| ATP5H | Directly related to OXPHOS reaction | Leyva et al., 2003 | [6] |
| ATPA | differential PTM after exposure to NOS | Sultana et al., 2006 | [5] |
| ECH1 | increased expression upon H_2_O_2_ stress | Kim et al 2011 | [7] |
| ECHS1 | co-regulated with ROS induced proteins | Severino et al., 2011 | [8] |
| EEF1D | differential up-regulation upon hyperoxia | Kaindl et al., 2006 | [9] |
| EF1D | differential up-regulation upon hyperoxia | Kaindl et al., 2006 | [9] |
| ERP29 | altered expression upon ROS exposure | Stockwin et al., 2007 | [10] |
| GAPDH | altered interaction partners; thioylation after exposure to H_2_O_2_  differential PTM after exposure to NOS | Hwang et al., 2009  Sultana et al., 2006 | [11]  [5] |
| GLUD1 | co-regulated with ROS induced proteins | Severino et al., 2011 | [8] |
| GRP78 (HSPA5) | GRP78 knockdown enhances ROS production | Chang et al 2012 | [12] |
| HNRDL | no ox. ref. |  |  |
| HNRNPA2B1 | no ox. ref. |  |  |
| HNRNPK | increased phosphorylation upon H_2_O_2_ stress | Ostrowski et al., 2000 | [13] |
| HSPD1 | resistance to oxidative stress | Cabiscol et al., 2002 | [14] |
| PCBP1 | altered expression upon ROS exposure | Stockwin et al., 2007 | [10] |
| PGK1 | increased expression upon H_2_O_2_ exposure | Jang et al 2008 | [15] |
| PRDX2 | differential up-regulation upon hyperoxia | Kaindl et al., 2006 | [9] |
| PSMA1 | differential up-regulation upon hyperoxia | Kaindl et al., 2006 | [9] |
| PSME2 | increased expression upon H_2_O_2_ exposure | Pickering et al., 2010 | [16] |
| TAGLN2 | no ox. ref. |  |  |
| TPI1 | increased carbonylation upon ROS exposure | Joshi et al., 2010 | [17] |

ROS = Reactive oxygen species; NOS = Nitric oxidative stress; PTM = Posttranslational modification;

no ox. ref. = No reference was found to experimentally associate this gene with oxidative stress metabolism.

### Additional References

1. Livak KJ, Schmittgen TD: **Analysis of relative gene expression data using real-time quantitative PCR and the 2(-Delta Delta C(T)) Method**. *Methods* 2001, **25**:402-408.
2. Felder B, Radlwimmer B, Benner A, Mincheva A, Tödt G, Beyer KS, Schuster C, Bölte S, Schmötzer G, Klauck SM, Poustka F, Lichter P, Poustka A: **FARP2, HDLBP and PASK are downregulated in a patient with autism and 2q37.3 deletion syndrome**. *Am J Med Genet A* 2009, **149**:952-959.
3. Poon HF, Vaishnav RA, Getchell TV, Getchell ML, Butterfield DA: **Quantitative proteomics analysis of differential protein expression and oxidative modification of specific proteins in the brains of old mice**. *Neurobiol Aging* 2006, **27**:1010-1019.
4. Sultana R, Perluigi M, Newman SF, Pierce WM, Cini C, Coccia R, Butterfield DA: **Redox proteomic analysis of carbonylated brain proteins in mild cognitive impairment and early Alzheimer's disease**. *Antioxid Redox Signal* 2010, **12**:327-336.
5. Sultana R, Poon HF, Cai J, Pierce WM, Merchant M, Klein JB, Markesbery WR, Butterfield DA: **Identification of nitrated proteins in Alzheimer's disease brain using a redox proteomics approach**. *Neurobiol Dis* 2006, **22**:76-87.
6. Leyva JA, Bianchet MA, Amzel LM: **Understanding ATP synthesis: structure and mechanism of the F1-ATPase (Review)**. *Mol Membr Biol* 2003, **20**:27-33.
7. Kim J, Kim E, Kim H, Yang J, Hwang G, Kim C: **Proteomic and metabolomic analysis of H2O2-induced premature senescent human mesenchymal stem cells**. *Exp Gerontol* 2011, **46**:500-510.
8. Severino V, Locker J, Ledda-Columbano GM, Columbano A, Parente A, Chambery A: **Proteomic characterization of early changes induced by triiodothyronine in rat liver**. *J Proteome Res* 2011, **10**:3212-3224.
9. Kaindl AM, Sifringer M, Zabel C, Nebrich G, Wacker MA, Felderhoff-Mueser U, Endesfelder S, Hagen M von der, Stefovska V, Klose J, Ikonomidou C: **Acute and long-term proteome changes induced by oxidative stress in the developing brain**. *Cell Death Differ* 2006, **13**:1097-1109.
10. Stockwin LH, Bumke MA, Yu SX, Webb SP, Collins JR, Hollingshead MG, Newton DL: **Proteomic analysis identifies oxidative stress induction by adaphostin**. *Clin Cancer Res* 2007, **13**:3667-3681.
11. Hwang NR, Yim S, Kim YM, Jeong J, Song EJ, Lee Y, Lee JH, Choi S, Lee K: **Oxidative modifications of glyceraldehyde-3-phosphate dehydrogenase play a key role in its multiple cellular functions**. *Biochem J* 2009, **423**:253-264.
12. Chang Y, Huang Y, Li Z, Chen C: **GRP78 knockdown enhances apoptosis via the down-regulation of oxidative stress and Akt pathway after epirubicin treatment in colon cancer DLD-1 cells**. *PLoS ONE* 2012, **7**:e35123.
13. Ostrowski J, Schullery DS, Denisenko ON, Higaki Y, Watts J, Aebersold R, Stempka L, Gschwendt M, Bomsztyk K: **Role of tyrosine phosphorylation in the regulation of the interaction of heterogenous nuclear ribonucleoprotein K protein with its protein and RNA partners**. *J Biol Chem* 2000, **275**:3619-3628.
14. Cabiscol E, Belli G, Tamarit J, Echave P, Herrero E, Ros J: **Mitochondrial Hsp60, resistance to oxidative stress, and the labile iron pool are closely connected in Saccharomyces cerevisiae**. *J Biol Chem* 2002, **277**:44531-44538.
15. Jang CH, Lee IA, Ha YR, Lim J, Sung M, Lee S, Kim J: **PGK1 induction by a hydrogen peroxide treatment is suppressed by antioxidants in human colon carcinoma cells**. *Biosci Biotechnol Biochem* 2008, **72**:1799-1808.
16. Pickering AM, Koop AL, Teoh CY, Ermak G, Grune T, Davies KJA: **The immunoproteasome, the 20S proteasome and the PA28alphabeta proteasome regulator are oxidative-stress-adaptive proteolytic complexes**. *Biochem J* 2010, **432**:585-594.
17. Joshi G, Aluise CD, Cole MP, Sultana R, Pierce WM, Vore M, St Clair DK, Butterfield DA: **Alterations in brain antioxidant enzymes and redox proteomic identification of oxidized brain proteins induced by the anti-cancer drug adriamycin: implications for oxidative stress-mediated chemobrain**. *Neuroscience* 2010, **166**:796-807.
